# Soy moratorium impacts on soybean and deforestation dynamics in Mato Grosso, Brazil

**DOI:** 10.1371/journal.pone.0176168

**Published:** 2017-04-28

**Authors:** Jude H. Kastens, J. Christopher Brown, Alexandre Camargo Coutinho, Christopher R. Bishop, Júlio César D. M. Esquerdo

**Affiliations:** 1 Kansas Applied Remote Sensing Program, Kansas Biological Survey, University of Kansas, 2101 Constant Ave., Lawrence, KS, United States of America; 2 Department of Geography and Atmospheric Science, University of Kansas, 1475 Jayhawk Blvd., 223, Lawrence, KS, United States of America; 3 Embrapa Informática Agropecuária, Av. André Tosello, n° 209, Campinas, SP, Brazil; University of Maryland at College Park, UNITED STATES

## Abstract

Previous research has established the usefulness of remotely sensed vegetation index (VI) data from the Moderate Resolution Imaging Spectroradiometer (MODIS) to characterize the spatial dynamics of agriculture in the state of Mato Grosso (MT), Brazil. With these data it has become possible to track MT agriculture, which accounts for ~85% of Brazilian Amazon soy production, across periods of several years. Annual land cover (LC) maps support investigation of the spatiotemporal dynamics of agriculture as they relate to forest cover and governance and policy efforts to lower deforestation rates. We use a unique, spatially extensive 9-year (2005–2013) ground reference dataset to classify, with approximately 80% accuracy, MODIS VI data, merging the results with carefully processed annual forest and sugarcane coverages developed by Brazil’s National Institute for Space Research to produce LC maps for MT for the 2001–2014 crop years. We apply the maps to an evaluation of forest and agricultural intensification dynamics before and after the Soy Moratorium (SoyM), a governance effort enacted in July 2006 to halt deforestation for the purpose of soy production in the Brazilian Amazon. We find the pre-SoyM deforestation rate to be more than five times the post-SoyM rate, while simultaneously observing the pre-SoyM forest-to-soy conversion rate to be more than twice the post-SoyM rate. These observations support the hypothesis that SoyM has played a role in reducing both deforestation and subsequent use for soy production. Additional analyses explore the land use tendencies of deforested areas and the conceptual framework of horizontal and vertical agricultural intensification, which distinguishes production increases attributable to cropland expansion into newly deforested areas as opposed to implementation of multi-cropping systems on existing cropland. During the 14-year study period, soy production was found to shift from predominantly single-crop systems to majority double-crop systems.

## Introduction

Previous studies have successfully applied vegetation index data from the Moderate Resolution Imaging Spectroradiometer (MODIS) to the study of agriculture, deforestation, and land change dynamics at the regional scale in the Brazilian Amazon, especially in the state of Mato Grosso (MT), which is one of the most dynamic agricultural frontiers in the world. Land cover (LC) datasets produced from MODIS have been used to study a wide range of human and environmental dynamics including land change impacts on biogeochemical cycling [1,2], fire frequency [3,4], and water quality [5,6]; physical geographic limits on agricultural practices [7–9]; regional socio-economic conditions [10,11]; and relationships among agricultural intensification, deforestation, and conservation [5,12–17].

Predominantly through LC classification, MODIS data also have been used in assessments of policy and governance efforts such as the “Soy Moratorium” (hereafter SoyM) designed to stem deforestation [18–26]. SoyM is an ongoing effort supported by environmental organizations and large agribusiness companies that involves a pledge not to purchase soy from areas deforested in the Amazon biome after July 24, 2006 [27]. In May 2016, SoyM was renewed indefinitely. MT accounts for approximately 85% of the soy grown in the Amazon biome [24].

Some of the most relevant research related to soy production has focused on the fate of deforested lands to determine the extent that deforestation is driven by soy production. The studies rely on data from the Program for the Estimation of Deforestation in the Brazilian Amazon (PRODES; http://www.obt.inpe.br/prodes/index.php) produced by Brazil’s National Institute for Space Research (INPE) to identify what areas of the Brazilian Amazon have been deforested each year going back to 1988 but with the most detailed data available beginning in 2000. Researchers typically then use MODIS data to classify post-deforestation lands to determine whether cropland or pasture replaced forests. In [17] it is reported that crop production had become a significant factor in deforestation because an increasing amount of cropland was replacing forests. The authors also report a correlation between increased cropland area and soy price, implying that increasing demand for soy had a role in causing deforestation. In a related study [16], this trend is reassessed with similar methods, and it is concluded that deforestation for soy production decoupled after the 2007 crop year (CY2007 = Aug 06 –Jul 07) based on a comparison between soy profitability and deforested land use. The authors describe a number of national and state government-led initiatives that could explain the drop in deforestation and subsequent decoupling with soy production, and they also mention industry-led initiatives such as SoyM as a potential factor in diminishing incentives to deforest for soy production. In [24] the authors argue that the SoyM should be renewed (which it eventually was) and extended spatially to the Cerrado (savanna) biome, based on their analysis of property-level impacts. Post-deforestation land use analysis using MODIS indicated a sharp decrease in deforestation for soy production, which they attribute in part to SoyM.

The present study contributes to the pursuit of policy-relevant research on agriculture and deforestation dynamics in the Amazon using remotely-sensed satellite data and GIS analyses. Building from our previous class separability study [28], we produce a 14-year LC time series for MT with a class structure that includes multiple soy and cotton classes in addition to others. By contrast, mapping efforts used in [17] and [16] did not seek to distinguish the type of cropland detected with MODIS, making the necessary assumption that cropland is most likely soy. This was avoided in [24] where a dataset was created following previous research [25, 26] that does claim to specifically map soy, but this was only for the part of their analysis that is in the Amazon (humid forest) biome. Their Cerrado biome analysis was based on a separate dataset in which all identified cropland is assumed to be soy. Finally, INPE’s PRODES data, though an extremely valuable research tool, is not without ambiguities and difficulties in its use. It is essential to describe how one processes the PRODES data to ensure results can be reproduced, as there has been a substantial lack of consistency among PRODES-based deforestation annual time series that have been independently developed for use in previous studies (S1 Fig).

The key outcomes of this work are three-fold. (1) Results from our PRODES-based deforestation analysis are more consistent with a large, direct SoyM effect on Amazon deforestation decline in MT than has been previously reported. (2) We create an updateable 14-year LC map set for MT and make it available for others to use. (3) We provide a detailed assessment of basic soy and deforestation dynamics in MT with attention to SoyM impacts.

For map production, we use downloadable MODIS Normalized Difference Vegetation Index (NDVI) time series data [28] and a random forest (RF) classification model [29]. The RF model is developed using a farmer interview-based ground reference dataset that is unprecedented in its spatial and temporal coverage, with minimal reliance on the less rigorous technique of visual interpretation of high resolution imagery to generate reference data. Following the image classification, INPE forest and sugarcane map data are overlaid to provide additional class detail. Model and map accuracy are examined using traditional metrics, probabilistic methods, federal crop statistics, and a spatially extensive, multi-year roadside dataset. We then utilize the map set to examine impacts of SoyM on deforestation and forest-to-soy conversion, to look at post-deforestation land use tendencies, and to examine the degree to which increased agricultural production in MT is being driven by conversion of forest to cropland (*horizontal intensification*) as compared to elevating production on existing cropland (*vertical intensification*; adapted from [28]).

## Study area

**Fig 1:**
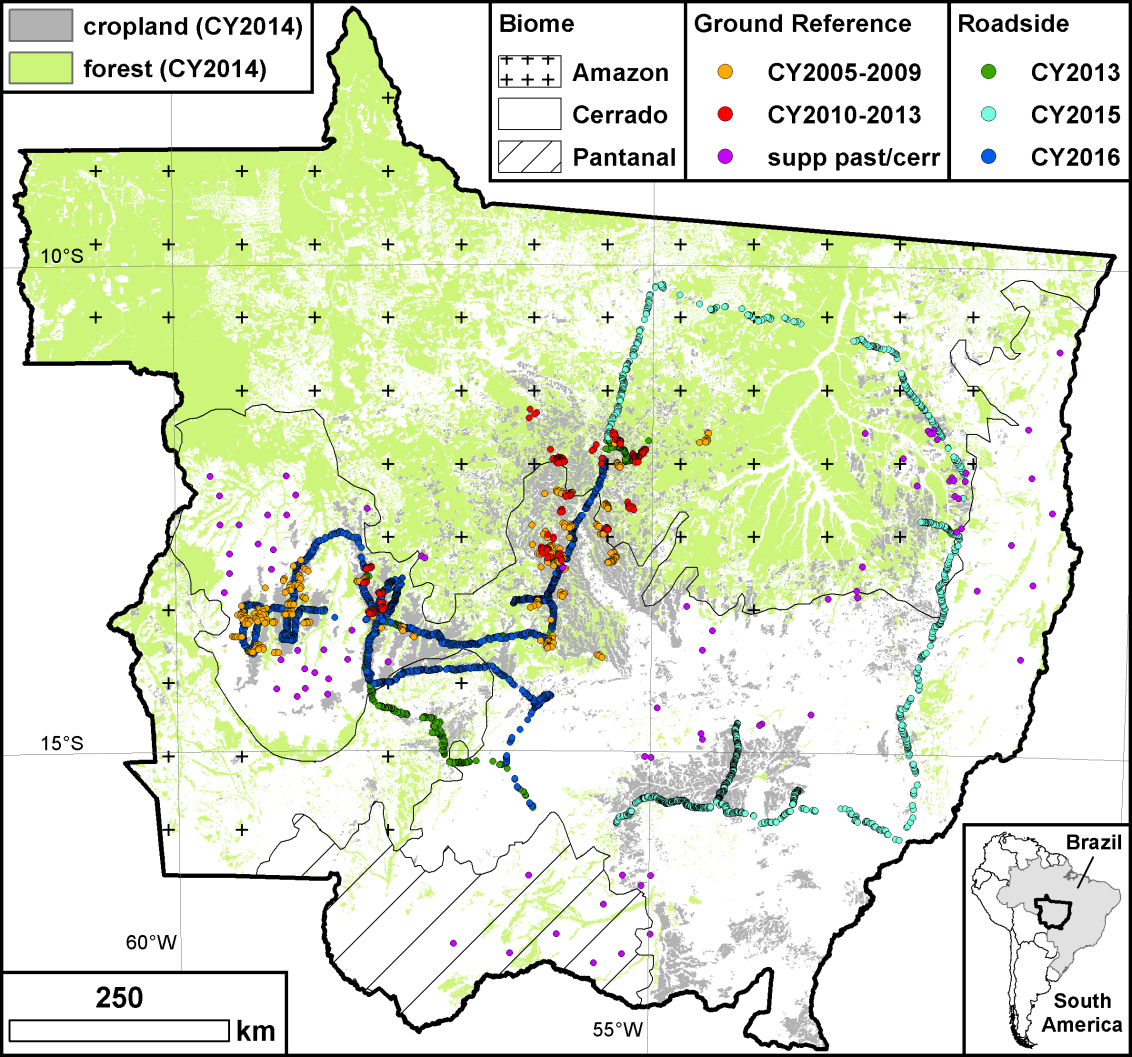
Study area (Mato Grosso). Forest and cropland geography as of CY2014 are shown along with biome boundaries and the locations of the ground reference and roadside data points.

MT covers approximately 904,000 km^2^ and is located in the center of the South American continent (Fig 1). Three official biomes comprise MT: the Pantanal wetland in the southwest (61,000 km^2^), the humid tropical forests of the Amazon in the north (484,000 km^2^), and the Cerrado (360,000 km^2^), a tropical savanna that extends from east to west through the center of the state. MT experiences a hot, semi-humid to humid climate (Koppen Aw), with a marked dry season from May through October.

Much of the state’s soils are old, deep, and nutrient poor. With inputs of fertilizer and lime, well adapted seed varieties, and favorable market conditions in recent years, MT increasingly has become a major source of agricultural production within Brazil, already a recognized agricultural superpower. According to official statistics published by the Brazilian Institute of Geography and Statistics (IBGE), during the crop year that ended in 2014, MT produced 28%, 21%, and 54% of Brazilian soybeans, corn, and cotton, respectively, making it the most productive Brazilian state for each of these major crops. The practice of increasing productivity through double cropping (sequentially growing and harvesting two commercial crops per year on the same land) has become increasingly prevalent across the region since 2000, where a second crop (called *safrinha* in Brazil) (predominantly corn) is planted after soy.

## Data and methods

Production of the 14-year (CY2001-2014) land cover time series for MT required a number of datasets. MODIS NDVI provided the independent variables to use for RF model development, while ground reference data from two field campaigns and a supplemental data development exercise provided the dependent variables necessary for model training, including crop type and pasture/cerrado data from CY2005-2013. Roadside data from three field campaigns representing CY2013, CY2015, and CY2016 were used for model and map performance evaluation. INPE PRODES data provided forest cover information for CY2001-2014. INPE Canasat data ([30]; http://www.dsr.inpe.br/laf/canasat/en/) provided sugarcane cover information for CY2003 and CY2005-2014. Static urban and water layers were obtained from IBGE.

### MODIS NDVI data

250-m, 16-day composite MOD13Q1 MODIS NDVI data covering MT for CY2001-2015 were downloaded from the Land Processes Distributed Active Archive Center (LP DAAC; https://lpdaac.usgs.gov/data_access). The data were reprojected from the native MODIS sinusoidal coordinate system to UTM Zone 21S (WGS84) with 240-m pixel size (5.76 ha/pixel) and a (0,0) registration coordinate. With 23 images/year (which we treat as regularly spaced) and using the composite period that begins on 28-July as the first image of the crop year, this resulted in a MT NDVI time series spanning 15 years and consisting of 345 samples. Data from CY2015 were used exclusively for LC trajectory examination during final map preparation for CY2014 (described in section F in S1 Supporting Information), but CY2015 is not included with the final map set due to the unavailability of required ancillary data at the time of the analysis. Additional MODIS NDVI data were acquired for CY2016 to use, along with the CY2015 MODIS data, for independent assessment of the RF model against roadside data corresponding with these crop years. We use NDVI rather than the Enhanced Vegetation Index (EVI) because NDVI is a simpler index, and our past research has revealed little difference in classification efficacy between the two datasets [28].

### Ground reference data and roadside data

In October 2009 and again in October 2013, research team members organized meetings with producers in key agricultural areas of MT and conducted a series of farmer interviews. Following the methodology explained in [31], each of these efforts produced a set of field boundary polygons with annual land cover information from recent crop years (Set 1: 2005–2009, 415 polygons; Set 2: 2010–2013, 191 polygons; see Fig 1 for sample locations). Original land cover designations were recoded to one of five possible classes [28]: (1) pasture/cerrado; (2) soy-single (single crop soybeans, possibly followed by a cover crop); (3) soy-double (double crop soybeans, or soybeans followed by a commercial crop, excluding cotton); (4) cotton; and (5) soy-cotton. The relatively few samples not fitting into one of these bins were discarded. According to IBGE statistics, crop classes (2)-(5) accounted for more than 95% of MT agricultural land area in CY2014.

**Fig 2:**
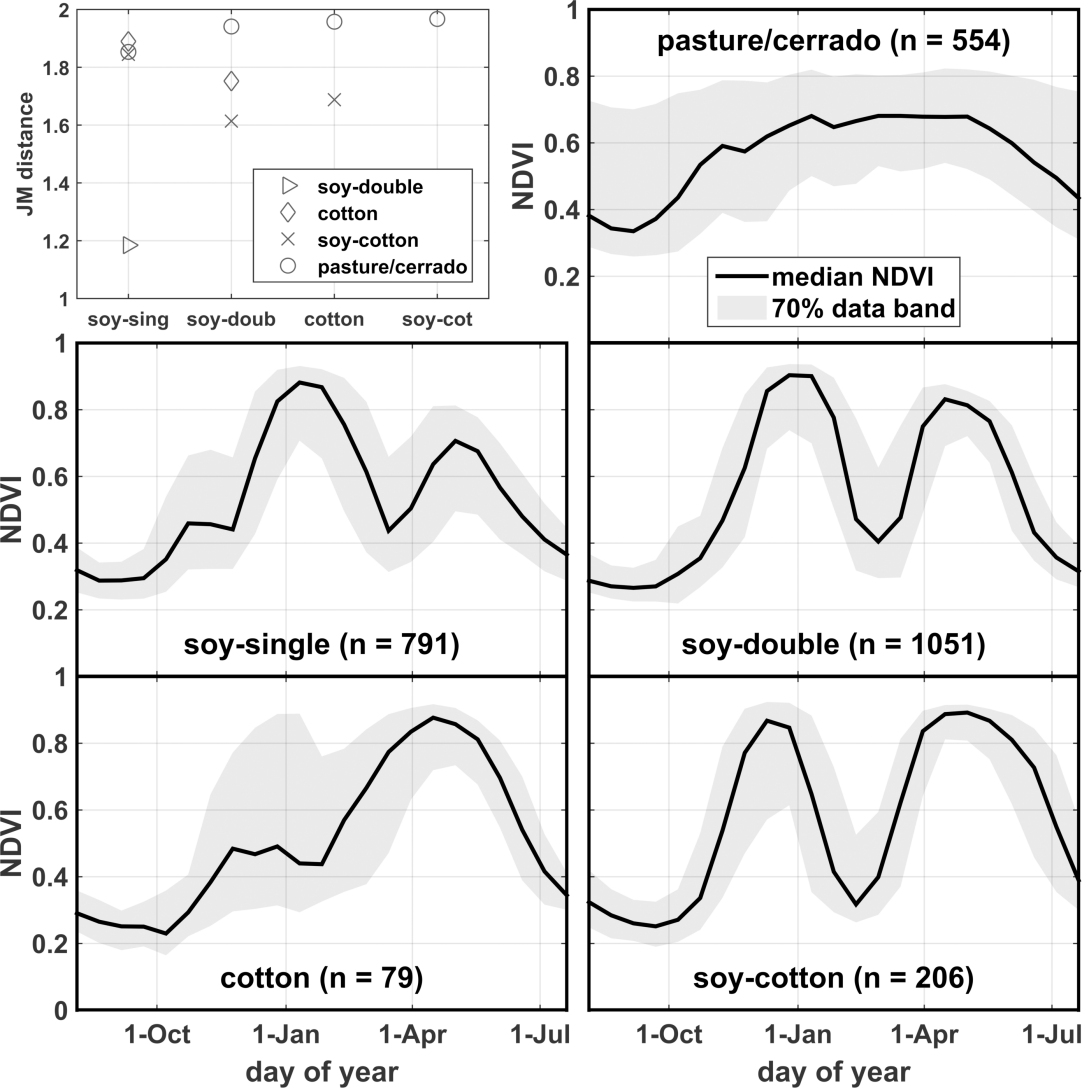
NDVI profile statistics. Ground reference data MODIS NDVI profile statistics are shown for the mapped classes. Pairwise Jeffries-Matusita (JM) distance statistics are shown in the upper left panel, which provide an indication of class separability (JM distance = 0 if classes are completely inseparable, JM distance = 2 if classes are completely separable).

Due to the crop-production focus of the meetings, the pasture/cerrado class was severely underrepresented in the polygon dataset, and thus a supplemental pasture/cerrado ground reference dataset was developed using high resolution imagery [12] to increase sample size and to round out statewide representativeness of the pasture/cerrado data in support of the RF modeling effort. See crop class counts in Table 1 and accompanying profile plots and separability statistics in Fig 2. Details regarding ground reference data preparation and supplemental pasture/cerrado data acquisition are described in sections B and C in S1 Supporting Information. Collectively, we refer to the data from the two farmer interview campaigns and the supplemental pasture/cerrado data development efforts as the “ground reference data” (S1 Dataset).

**Table 1:** Ground reference data sample counts.

Crop Year	Pasture/	Soy-	Cotton	Soy-	Soy-	TOTAL
	Cerrado	Single		Double	Cotton	
2005	104	148	5	94	21	372
2006	103	189	13	107	21	433
2007	106	128	9	151	21	415
2008	104	111	17	185	12	429
2009	107	122	12	180	10	431
2010	8	23	2	58	23	114
2011	7	37	9	76	36	165
2012	7	16	10	80	44	157
2013	8	17	2	120	18	165
TOTAL	554	791	79	1051	206	2681

During July 29-August 2, 2013, research team members conducted a roadside survey through key growing regions of MT, visually identifying pasture/cerrado and agricultural fields from CY2013 and registering them on a GIS/GPS-enabled tablet. Stopping points generally were selected randomly as a function of travel time/distance from the previous point, with some consideration for accessibility as needed. For agricultural fields, stubble or residue from the most recent vegetative cover was identified, and if non-soy, then the ground was examined for stubble/residue from a preceding soy crop. Cases where on-the-ground evidence for a preceding soy crop was not readily apparent were subjected to a MODIS CY2013 vegetation index profile examination using the Temporal Vegetation Analysis System MODIS visualization tool (SATVeg; www.satveg.cnptia.embrapa.br). If the profile exhibited pronounced bimodality suggestive of a preceding soy crop (see soy class profiles in Fig 2), then soy was ascribed as preceding the identified most recent vegetative cover. Two similar roadside surveys were conducted gathering points for CY2015 (October 5–9, 2015) and CY2016 (May 21–25, 2016). While the latter two datasets fall just outside our study period, they are nonetheless useful for examining the spatiotemporal generality of the RF model. Collectively we refer to all of the data from the roadside campaigns as the “roadside data” (S2 Dataset). See Fig 1 for roadside data locations and Table 2 for sample counts by year and by class.

**Table 2:** Roadside data sample counts.

Crop Year	Pasture/	Soy-	Cotton	Soy-	Soy-	TOTAL
	Cerrado	Single		Double	Cotton	
2013^a^	135	82	0	427	71	715
2015	339	186	34	308	57	924
2016	228	189	12	755	258	1442
TOTAL	702	457	46	1490	386	3081

^a^104 sugarcane points also were collected and are included in the Supplementary Information (S2 Dataset)

### INPE PRODES data

We prepared annual forest cover layers for CY2001-2014 using INPE PRODES data, which is a polygon coverage available from http://www.dpi.inpe.br/prodesdigital/prodes.php (download date: 17-Sep-15). The PRODES dataset has tracked deforestation in Brazil since 1988 and is annually revised and updated by INPE primarily using Landsat imagery. Date-specific (year and day-of-year) deforestation information, which indicates the acquisition date for the satellite image where deforestation for a particular polygon was first detected, extends back through calendar year 2000. The PRODES data included forest and deforested polygons. PRODES data preparation and processing details are provided in section D in S1 Supporting Information. To maintain consistency with the MODIS time series, we used the same cutoff date (28-July) as the start of a crop year.

If a polygon was indicated to be deforested during a specific crop year, then this polygon remains as forest in our land cover map for that crop year and becomes opened up for non-forest classification in subsequent crop years. For example, with this definition any deforestation occurring in CY2007 (the first crop year after implementation of SoyM) first shows up as non-forest in the map for CY2008, and the difference in forest area between the maps from CY2007 and CY2008 represents the total area that was deforested during CY2007. During the 14-year study period, 87% of the total deforested area occurred within the Amazon biome, with annual values ranging 83–91%.

### INPE Canasat data

INPE Canasat data, which were kindly provided by the dataset stewards for the development of this work, were used to define annual sugarcane coverages for MT for CY2003 and CY2005-2014. Coverages for CY2001, CY2002, and CY2004 were developed using Canasat data from CY2003 and CY2005. Details of this backfill process are described in section E in S1 Supporting Information.

### Random forest model

An overview of RF and its use for LC classification is provided in [32], highlighting a number of strengths of this modeling approach (e.g. no assumptions about data distributions, nonlinear, robust to outliers and noise). Anecdotal justification for our decision to use RF is provided in section A in S1 Supporting Information. In this study, RF model development is based on the polygonal ground reference dataset, utilized in a GIS framework that incorporates the pure pixel approach [28, 33–35] in which NDVI values from a single interior pixel with a relatively noise-free NDVI profile are extracted from each homogeneously managed farm field polygon for years in which cropping practices are known.

MATLAB® software was used for RF model development, specifically the ‘treebagger’ function. A RF model consists of many small decision trees (DT; 1000 in this case) and a simple plurality voting strategy for evaluation when presented with a data observation of the independent variables (the MODIS profile time series values in this case). Each component DT is developed independently from a random subset of the candidate predictors (5 out of 23 MODIS periods in this case) and a bootstrap replica of the training data. Samples not used for training a particular DT are referred to as ‘out-of-bag’ (OOB) samples for that DT.

### Processing steps for map development

The following list summarizes the processing steps used to create the final CY2001-2014 MT LC map set:

1. Create spatially smoothed RF output:
  - Apply 5-class RF model to MODIS NDVI image stacks
  - Apply one-pass, 3-by-3 modal spatial filter to all years (to despeckle)
2. Apply overlays:
  - Overlay annual PRODES data
  - Overlay annual Canasat data
  - Overlay static urban layer
  - Overlay static water layer
3. Identify and repair spatial and temporal data irregularities (for details, see section F in S1 Supporting Information)
  - Identify and repair PRODES data commission and omission anomalies
  - Identify and repair illogical pixel LC trajectories

## Results and discussion

**Fig 3:**
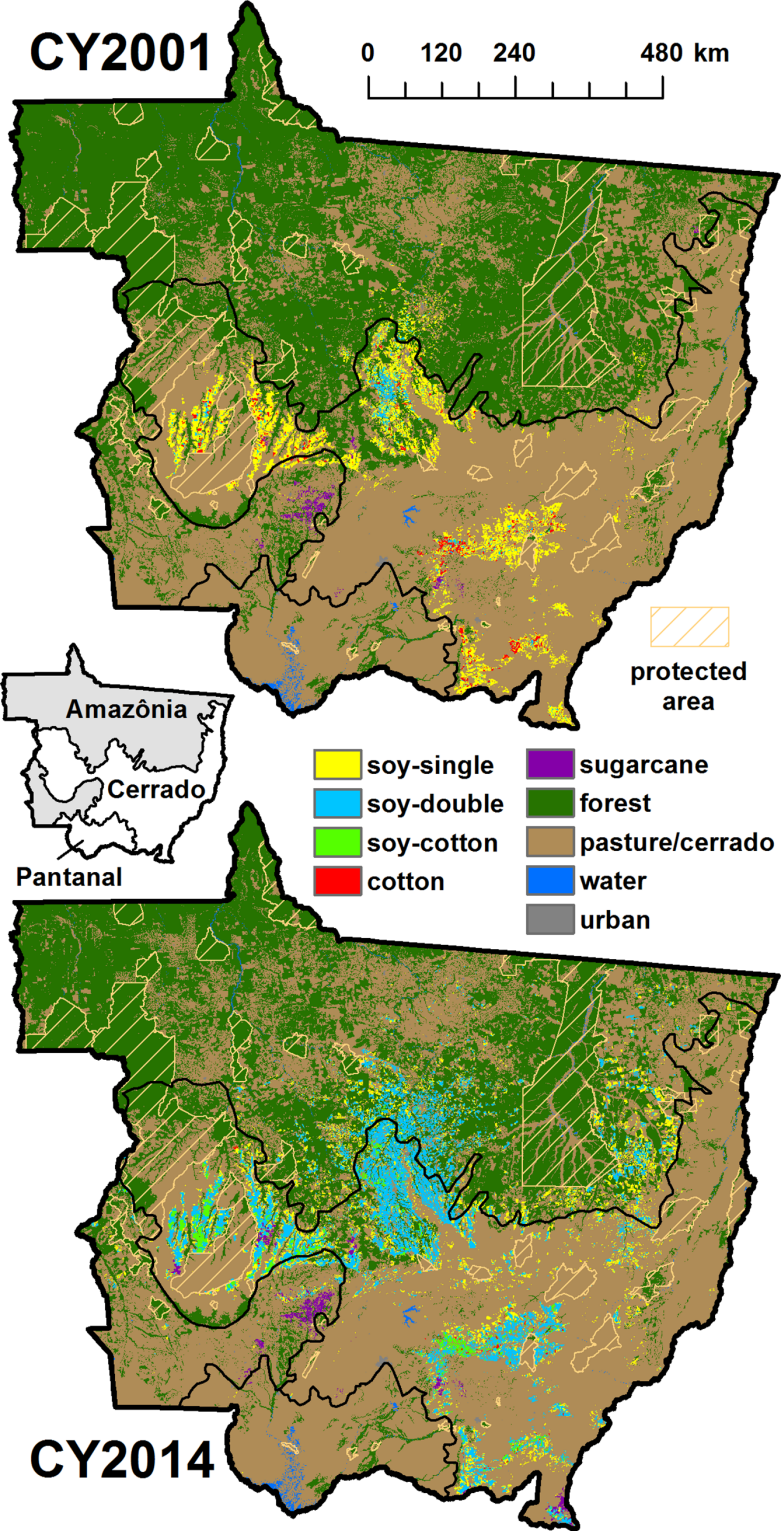
Mato Grosso land cover for crop years 2001 and 2014. Final LC maps are shown for study period endpoints CY2001 and CY2014. Biome boundaries, which are overlaid on the LC maps, are labeled on the inset map. Protected areas (indigenous reserves) are also shown on the LC maps.

LC maps for CY2001 and CY2014 are shown in Fig 3, the endpoints of our study period. Table 3 provides annual class area totals used for assessing state-level LC dynamics. In this section the outcomes from several evaluations are presented. First, RF model accuracy and map performance are examined using multiple approaches. Next, LC change dynamics are examined, with attention to SoyM, post-deforestation land use tendencies, and agricultural intensification.

**Table 3:** Land cover area totals for MT.

	Mapped Area (km^2^)^a^							Area Summaries			
Crop	Forest	Pasture/	Soy-	Soy-	Cotton	Soy-	Sugar-	All Soy^b^	All	All	Deforest^e^
Year		Cerrado	Single	Double		Cotton	cane		Cotton^c^	Cropland^d^	
2001	388,674	472,490	29,889	3,289	3,013	110	1,588	33,288	3,123	37,888	16,821
2002	371,852	487,421	28,652	7,123	1,919	404	1,681	36,179	2,323	39,779	5,612
2003	366,240	488,265	31,671	8,977	1,796	396	1,707	41,044	2,192	44,547	9,114
2004	357,126	497,880	24,600	13,705	2,549	1,196	1,995	39,501	3,745	44,045	14,639
2005	342,488	499,077	40,546	11,764	2,413	733	2,031	53,043	3,146	57,487	7,816
2006	334,671	504,907	40,731	13,492	1,614	1,506	2,131	55,728	3,120	59,473	9,312
2007	325,359	517,584	29,639	20,532	2,477	1,095	2,366	51,265	3,572	56,108	2,457
2008	322,902	513,626	32,316	23,157	2,894	1,518	2,638	56,992	4,412	62,524	2,918
2009	319,984	516,782	33,031	23,315	1,769	1,360	2,810	57,706	3,129	62,285	3,447
2010	316,536	517,332	33,373	26,591	782	1,657	2,782	61,620	2,439	65,184	1,175
2011	315,362	514,961	34,765	26,532	2,282	2,330	2,820	63,627	4,612	68,729	1,054
2012	314,308	516,372	22,752	36,382	1,888	4,477	2,873	63,611	6,365	68,372	725
2013	313,582	509,241	29,039	40,358	489	3,320	3,023	72,716	3,808	76,228	940
2014	312,642	504,525	29,208	43,843	466	5,383	2,984	78,434	5,849	81,884	1,006

^a^Total area = 904,226; Water (4,222) and Urban (953) areas were held constant

^b^Sum of Soy-Single, Soy-Double, and Soy-Cotton

^c^Sum of Cotton and Soy-Cotton

^d^Sum of Soy-Single, Soy-Double, Cotton, Soy-Cotton and Sugarcane

^e^{Deforest during ‘Year’} = {Forest from ‘Year’}–{Forest from ‘Year+1’}; 2015 Forest area = 311,636

### Evaluating RF model accuracy and map performance

We examine RF model accuracy and map performance using four assessments. First, to examine model efficacy, we present OOB accuracy results for the model. Second, to examine probabilistic mapping error, we rescale the OOB results to reflect map proportions, using IBGE crop statistics to support the evaluation and to provide an alternative perspective. Third, to examine the spatiotemporal generality of the model, we evaluate classification results using the roadside data. Fourth, to examine crop-specific agreement with federal estimates, we compare annual state-level soybean and cotton area totals from the classified maps to IBGE crop statistics.

#### OOB error

One convenience of RF modeling is that an unbiased estimate for model accuracy can be produced using the OOB samples, which, for our RF setup, essentially is equivalent to the expected value of a stratified random cross validation with about 2/3 of the data used for training and 1/3 set aside for validation [29,32]. To compute OOB error, the RF model is applied to the training data in a manner such that only OOB samples are evaluated for each particular component DT, which ensures that all predictions from each DT, and thus from the RF model as a whole, will be out-of-sample.

OOB results from the RF model used in this study produced a Kappa statistic of 0.71 and an overall accuracy of 79% (Table 4). User’s accuracy values for all five classes are 70% or greater. As a check on the stability of the overall accuracy estimate, 30 independent, replicated RF optimizations and OOB evaluations were performed, and the overall accuracies were found to be highly stable (either 79% or 80% in all cases). As a final test, we evaluated the RF modeling procedure in a rigorous one-year holdout cross validation [28], whereby an RF model was developed using eight years of ground reference data and applied to the withheld year, repeating this process nine times such that each year served as a holdout year and then aggregating all of the holdout year results. We repeated this exhaustive cross validation procedure 30 times, obtaining an overall accuracy of 77% in every case.

**Table 4:** RF model OOB accuracy. The confusion matrix and traditional classification accuracy statistics are given for the OOB results produced using the RF model.

		Reference Class					
		Past/Cerr	Soy-Sing	Cotton	Soy-Doub	Soy-Cot	Total
Predicted	Pasture/Cerrado	507	42	6	13	2	570
	Soy-Single	41	591	6	154	7	799
	Cotton	1	3	39	1	12	56
	Soy-Double	5	154	21	869	63	1112
	Soy-Cotton	0	1	7	14	122	144
	Total	554	791	79	1051	206	2681
User’s Accuracy		89%	74%	70%	78%	85%	
Producer’s Accuracy		92%	75%	49%	83%	59%	
Overall Accuracy		79%		Kappa		0.71	

#### Probabilistic mapping error

Combining all four modelled agricultural classes into a general ‘soy/cotton’ class, overall accuracy using the OOB results increases to 96% and Kappa to 0.88. These numbers reflect the RF model’s ability to classify sample data proportioned and distributed (with respect to NDVI profiles) similarly to the ground reference data. Alternatively, we can scale the OOB confusion matrix values to reflect mapped data proportions instead of sample proportions [36]. Pasture/cerrado comprises 90% of the classified pixels, so that class generally will dominate map accuracy assessments. To mitigate this problem, we excluded map data from the MT municipalities with the least soy/cotton (here defined as total soy area plus total cotton area, including a double counting of the soy-cotton class). This increased the soy/cotton proportion in the remaining mapped area and thus soy/cotton influence on map accuracy statistics. Specifically, for each map year, the 141 municipalities comprising MT were sorted by A = max(IBGE soy/cotton area, mapped soy/cotton area). IBGE annual total crop area estimates were obtained from http://www.sidra.ibge.gov.br. The municipalities with the least soy/cotton area (smallest A values) were sequentially excluded until their cumulative soy/cotton area (cumulative A) reached 10% of total A, an arbitrarily selected threshold chosen to balance soy/cotton area preservation with low soy/cotton area exclusion.

Excluded municipality counts ranged from 94 in CY2014 to 113 in CY2001. Pasture/cerrado fraction in the included municipalities was reduced to a 14-year total of 75%, with annual values ranging 73–80%. Using definitions from [36] to characterize map accuracy, the 14-year total *proportion correct* was estimated to be 91.2% (8.8% *total disagreement* = 7.7% *quantity error* + 1.1% *allocation error*) for a {pasture/cerrado, soy/cotton} two-class split. Proportion correct is equivalent to overall accuracy; quantity error reflects absolute over/under-mapping in excess of errors that hypothetically could be remedied through class swapping (allocation error).

In this two-class examination, it is readily determined from the proportion-adjusted confusion matrix that the 7.7% quantity error is meant to reflect an under-mapping of soy/cotton. With soy/cotton comprising 25% of the 14-year total examined area, this under-mapping would seem to suggest that our maps fail to represent 24% of MT soy/cotton (7.7/(25+7.7)), a substantial error indeed. However, statewide comparison between IBGE estimated soy/cotton area and mapped soy/cotton area provides evidence to the contrary, with 14-year total mapped soy/cotton area equal to 93% of the 14-year total IBGE soy/cotton area, implying a much more modest under-mapping error of just 7%. Thus we posit that the inferred statewide estimate of 24% under-mapped soy/cotton is greatly overstated, a consequence of ground reference sample characteristics and assumptions underlying the quantity error statistic. Had we considered the entire state (i.e. no municipality screening), the theoretical under-mapping error for soy/cotton is overstated more severely at 49%, illustrating the importance of exercising caution when making map accuracy considerations using probabilistic extrapolation. While estimating map accuracy in this manner generally is good practice, other more direct measures (comparison to widely accepted IBGE statistics in this case) can provide more reliable assessments when suitable ancillary data are available.

#### Independent roadside data assessment

While the previous section addressed the sample vs. map proportion issue with regard to estimating map accuracy, this section addresses the sample representativeness issue. The question is whether or not the ground reference data sample is spatiotemporally representative of statewide crop conditions and management that are reflected in MODIS NDVI profiles. During three different field campaigns, team members collected roadside data samples from the four main soy/cotton growing areas around the state [12], and here we assess RF model performance against these samples. Data from two of the campaigns (CY2015 and CY2016) were from years not represented in the ground reference dataset used for model construction and are thus temporally out-of-sample. The three roadside datasets should collectively represent the overall statewide spatial variability in class-specific NDVI profile distributions more completely than the ground reference dataset, which spatially was more confined (Fig 1).

More than 3000 roadside samples were collected. Land cover classifications from the RF model applied to the respective crop year were determined for each point and compared to the roadside class, resulting in a Kappa statistic of 0.78 and an overall accuracy of 85% (Table 5). The low producer’s accuracy for the cotton class could be attributable to its disproportionately small sample size relative to the ground reference data, and with most of the error accounted for by misspecification as soy-cotton, possibly also to model confusion caused by the frequently strong early-season signal seen in the MODIS profile distribution for cotton (Fig 2). 104 sugarcane points that were collected with the CY2013 dataset yielded user’s and producer’s accuracy values of 97% and 85%, respectively, which supports our reliance on the Canasat data.

**Table 5:** RF model accuracy assessment using the roadside data. The RF model was applied to the roadside data. Results are aggregated across all three years of data: CY2013 (n = 715), CY2015 (n = 924), CY2016 (n = 1442).

		Reference Class					
		Past/Cerr	Soy-Sing	Cotton	Soy-Doub	Soy-Cot	Total
Predicted	Pasture/Cerrado	653	64	6	28	2	753
	Soy-Single	32	312	0	140	3	487
	Cotton	1	0	11	2	2	16
	Soy-Double	15	81	3	1313	53	1465
	Soy-Cotton	1	0	26	7	326	360
	Total	702	457	46	1490	386	3081
User’s Accuracy		87%	64%	69%	90%	91%	
Producer’s Accuracy		93%	68%	24%	88%	84%	
Overall Accuracy		85%		Kappa		0.78	

Looking at the roadside locations across time, we can check if overall accuracy values are behaving as would be expected from one-year probability samples representative of the dynamic MT agricultural landscape. Specifically, one would expect overall accuracy to peak during the target year and generally decline backward and forward in time. Indeed, this is what we observe for all three years (Table 6), an outcome supporting the general spatiotemporal validity of the RF model across the MT study area.

**Table 6:** Overall accuracy of RF model class with the roadside data. Agreement should exhibit a clear peak at the target year (highlighted cells) as well as decay moving away from the target year, which is observed with all three roadside datasets.

	Overall Accuracy		
Crop Year	2013	2015	2016
2001	34%	45%	31%
2002	41%	47%	37%
2003	45%	50%	40%
2004	51%	51%	47%
2005	51%	51%	46%
2006	50%	53%	47%
2007	59%	58%	52%
2008	63%	58%	56%
2009	64%	60%	56%
2010	64%	64%	58%
2011	65%	63%	57%
2012	74%	68%	66%
2013	***86%***	73%	72%
2014	77%	75%	75%
2015	71%	***84%***	76%
2016	72%	76%	***84%***

#### Comparison to IBGE crop area statistics

**Fig 4:**
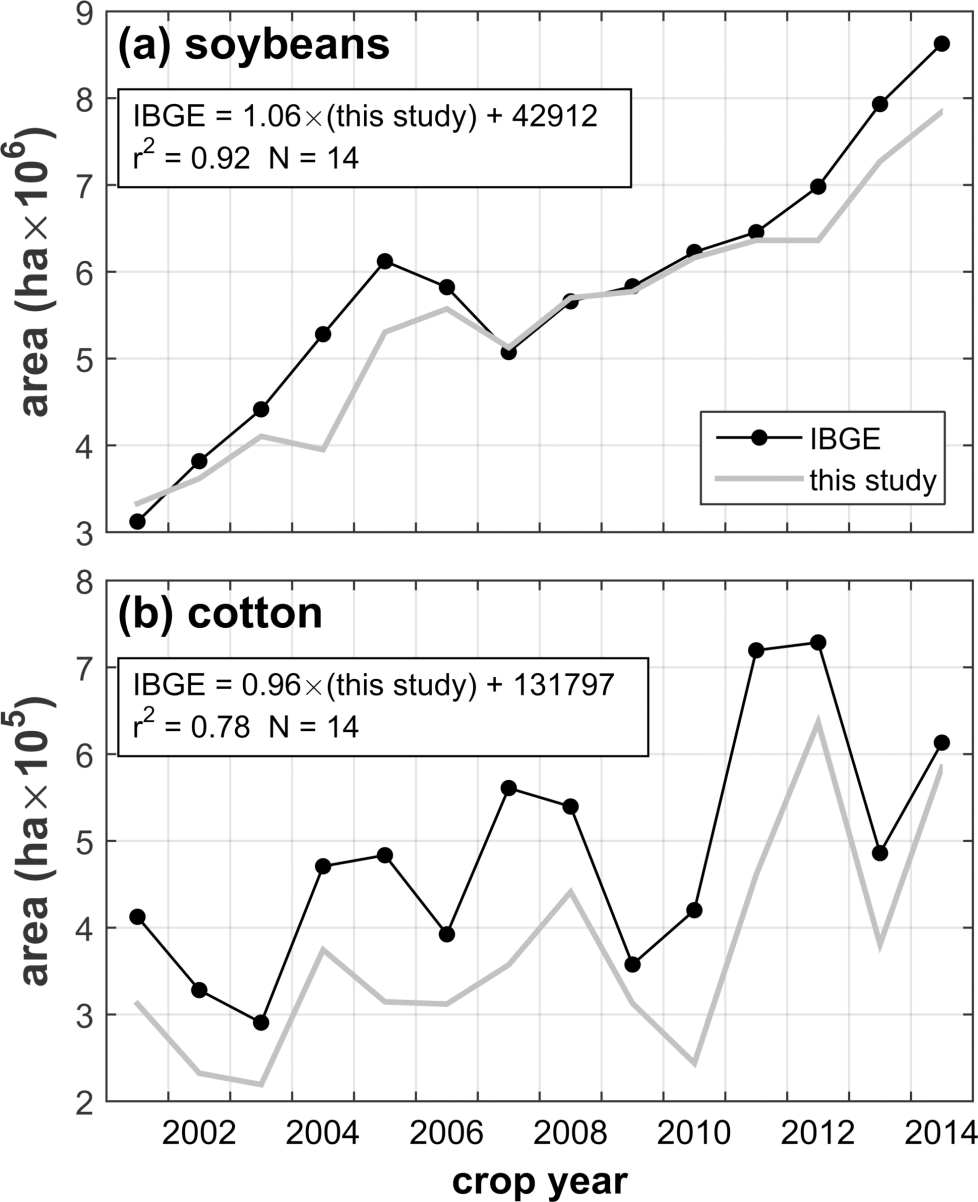
Map area totals compared to IBGE estimates. Total crop area from the LC maps is shown along with corresponding IBGE crop area statistics for (a) soybeans and (b) cotton. Linear regression equations and associated statistics are provided on the plots.

We compared IBGE crop areas for soybeans and cotton to the LC map totals across the study period (Fig 4). With reasonably high correlation coefficients and regression slope coefficients near one, much of the year-to-year variability (including the general uptrend) in both IBGE series is reflected in the map results. Additionally, with a relatively small intercept, the mapped soybean totals exhibit low bias. The same cannot be said for cotton, however, as the maps fairly consistently underestimate cotton area when compared to the IBGE statistics. While comparison of mapped crop area to statewide IBGE crop area estimates is not a direct reflection of spatial accuracy, as described earlier it does provide a simple and direct indicator of quantity error. One should expect a reasonable level of agreement between these values for reliable map sets. That temporal trends and fluctuations are found to coincide to a large degree provides further affirmation of the temporal generality of the RF model for the study area.

### Assessing LC change during CY2001-2014

The spatial and temporal coverage of our map set makes it applicable for a variety of assessments, which we illustrate with three analyses. The first assesses deforestation rates in relation to SoyM and land use tendencies of deforested areas. The second examines the increase in double cropping on soy cropland observed during the study period. The third analysis employs the conceptual framework of horizontal vs. vertical intensification to explore the relationship between agricultural intensification and deforestation.

#### Impacts of SoyM on deforestation rates and fates of deforested lands

**Fig 5:**
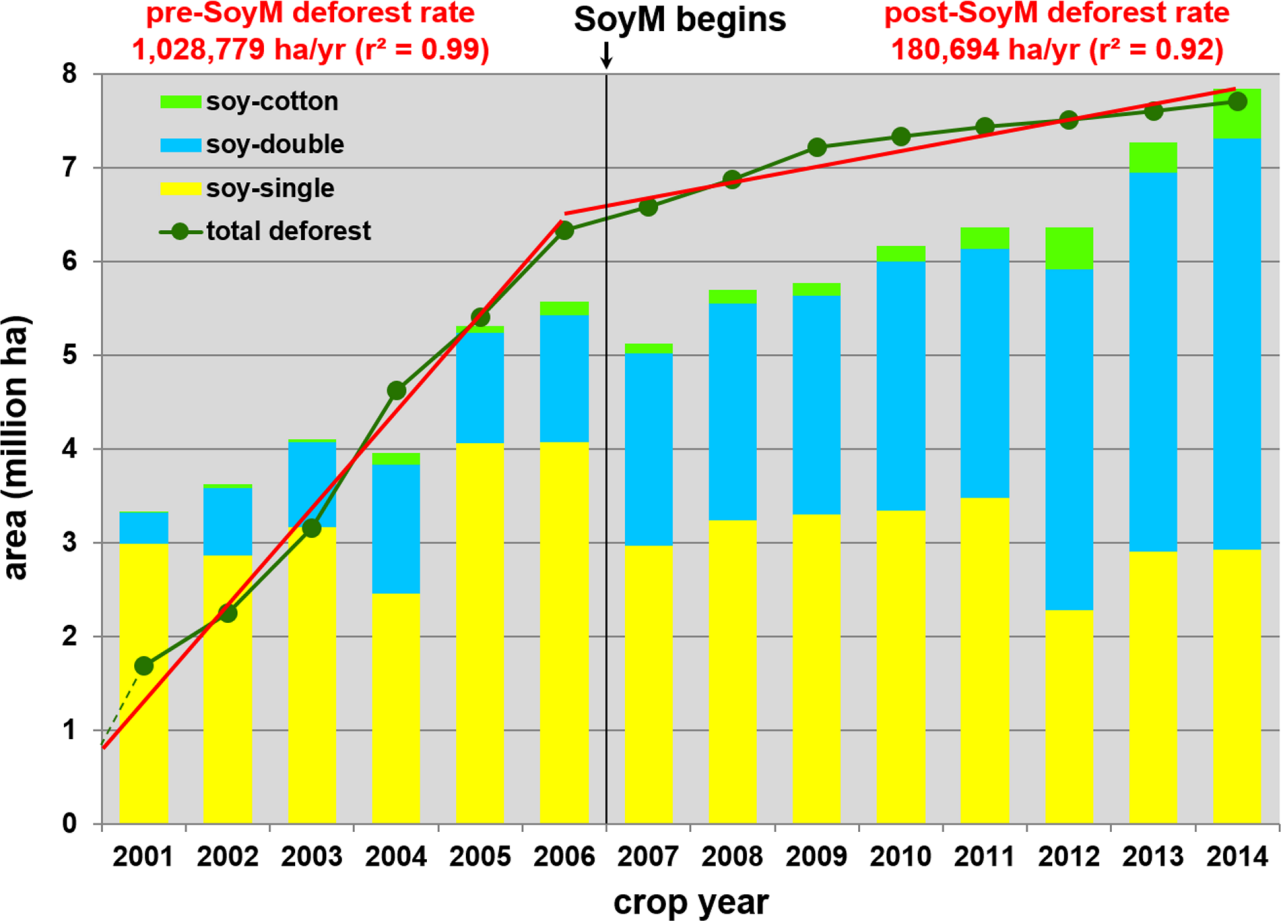
Map area totals for deforest and soy. Map-based areal data summaries for accumulated deforestation and annual soybeans are shown. Pre-SoyM and post-SoyM deforest trend lines are also depicted, with respective regression slopes provided in red text above the graph. A value of 0 was assigned to CY2000 to anchor the pre-SoyM total deforest trend line in the same manner that CY2006 is used to anchor the post-SoyM total deforest trend line

Accumulated deforested area (green line) is shown in Fig 5, which is the set of running sums of the last column of Table 3. The progression of total deforest values essentially reflects deforestation information provided in the PRODES dataset that we prepared for MT.

With SoyM taking effect between CY2006 and CY2007, the reduction in annual deforestation rate immediately after implementation is starkly evident, more pronounced than reported in either [16] or [23] (S1 Fig). The minimum pre-SoyM deforestation rate (561,185 ha/yr in CY2002) is more than 60% greater than the maximum post-SoyM deforestation rate (344,748 ha/yr in CY2009). Overall, relatively stable deforestation rates are observed during both the pre-SoyM and post-SoyM periods, with the post-SoyM trend line rate representing a reduction of the pre-SoyM trend line rate by a factor of 5.7 (Fig 5).

One important question surrounding deforested areas pertains to their subsequent land use, particularly for soy production. Using the LC map set, we calculated the post-deforestation land use practices for 1 to 11-year time lags following the crop year of deforestation with respect to use as pasture/cerrado, for soy production (including soy-single, soy-double, and soy-cotton), and for cotton (cotton and soy-cotton) or sugarcane production (Table 7). With a 1-year lag, the map results indicate that soy was planted on 2.8% of lands deforested during the previous crop year, whereas 97.1% ended up as pasture/cerrado. For greater lags, a steady increase in soy prevalence is seen up to about 9 years, where use for soy production peaks at 17.8%. The soy occurrence rate then decreases slightly for 10 and 11-year lags, with the drops partly offset by an increase in cotton/sugarcane proportions. Generally as lag increases, the number of years available for averaging declines, decreasing the stability of the larger lag results.

**Table 7:** Mean percent cover following deforestation. Conversion to soy increases with lag time, peaking at 9 years.

				Cotton
Lag	Crop Years	Pasture/	Soy	(2 classes)
(years)	Analyzed	Cerrado	(3 classes)	+
				Sugarcane
1	02–14	97.1	2.8	0.08
2	03–14	94.9	5.0	0.03
3	04–14	93.2	6.8	0.03
4	05–14	91.1	8.8	0.05
5	06–14	89.9	10.0	0.08
6	07–14	88.1	11.8	0.08
7	08–14	85.9	14.1	0.13
8	09–14	84.2	15.7	0.15
**9**	**10–14**	**82.2**	**17.8**	**0.19**
10	11–14	82.7	17.2	0.35
11	12–14	83.6	16.2	0.36

In addition to SoyM implementation coinciding with an abrupt decline in deforestation rate, we also found a decrease in forest-to-soy conversion when comparing pre-SoyM rates to post-SoyM rates. We can restrict the post-deforest land cover analysis just described to 1 to 5-year time lags and evaluate the outcomes exclusively using the pre-SoyM time period (CY2001-2006) and the post-SoyM time period (CY2007-2014). Results support the conclusion that the forest-to-soy conversion rate has declined in MT following implementation of SoyM (Table 8). Averaged across all five lag intervals, the post-SoyM forest-to-soy conversion rate decreased by a factor of 2.4 (9.4% down to 3.9%) compared to the pre-SoyM rate, with a decrease factor range of 1.9–3.2 across the different lags.

**Table 8:** Mean percent cover following deforestation, pre- vs. post-SoyM. Following implementation of SoyM, rate of conversion to soy was found to decrease for each of five examined lag intervals.

					Cotton
	Lag	Crop Years	Pasture/	Soy	(2 classes)
	(years)	Analyzed	Cerrado	(3 classes)	+
					Sugarcane
pre-SoyM	1	02–06	95.1	4.9	0.02
	2	03–06	91.8	8.1	0.04
	3	04–06	90.7	9.2	0.04
	4	05–06	89.5	10.5	0.06
	5	06	85.7	14.2	0.06
	**average**		**90.6**	**9.4**	**0.04**
post-SoyM	1	08–14	98.3	1.5	0.13
	2	09–14	97.1	2.9	0.03
	3	10–14	95.8	4.1	0.02
	4	11–14	94.4	5.6	0.03
	5	12–14	94.4	5.6	0.04
	**average**		**96.0**	**3.9**	**0.05**

#### Production intensification on soy cropland

The general increase in total soy area during the study period is accompanied by the increase in double cropping of soy with another commercial crop, including cotton (Fig 5). While single crop soy plantings remained fairly constant over the study period, soy cultivation in general increased from 3.3 Mha in CY2001 to 7.8 Mha in CY2014 (Table 3), which is an increase by a factor of 2.4. In terms of total area of commercial crops coming off land used for soy production (i.e. double counting soy-double and soy-cotton in area totals), the corresponding numbers are 3.7 Mha in CY2001 and 12.8 Mha in CY2014, which is an increase by a factor of 3.5. Another way of looking at this is in terms of average number of commercial crops per soy pixel, which increased from 1.1 in CY2001 to 1.6 in CY2014, indicating a shift of soy production from predominantly single-crop systems to majority double-crop systems.

### Horizontal intensification vs. vertical intensification

Though a large amount of forest land has been cleared during the study period and converted to cropland (horizontal intensification), it is also true that a large amount of pre-existing cropland has seen an increase in productivity in the form of multi-cropping (vertical intensification). We present a simple comparison to illustrate the different degrees to which soy-related agricultural intensification is occurring with respect to the horizontal and the vertical.

Considering that forest-to-soy conversion rate appeared to hit a maximum with a lag of nine years (Table 7), we define a horizontal intensification area to be the accumulated area deforested during CY2001-2005 and then calculate the fraction of this area that was classified as soy in CY2014. All of this deforested land (which is not restricted by SoyM) will have had at least nine years to be converted to soy (soy-single, soy-double, or soy-cotton). To define a comparable vertical intensification area, we consider all single crop soy plantings (soy-single) in CY2005 and compute the fraction of this area that has been converted to double crop soy plantings (soy-double or soy-cotton) in CY2014. With these definitions, the horizontal area comprises 5.4 Mha and the vertical area a comparable 4.1 Mha.

In CY2014, we find that 1.2 Mha of the horizontal area had been converted to soy, for a conversion fraction of 22%. In CY2014, we find that 2.5 Mha of the vertical area had been converted to soy-double or soy-cotton, for a conversion fraction of 61%. Based on this assessment, soy producers intensified production on existing soy cropland at 2.8 times the relative rate at which they expanded soy production to deforested areas. This direction of the intensification dynamic in MT is consistent with the ideals expressed by Norman Borlaug that increased production on existing cropland can relieve pressure to convert tropical forests for agriculture [37]. However, this simplistic assessment ignores location-specific factors such as agricultural production potential and market accessibility that could well affect the interpretation of this outcome [7,9].

## Conclusion

In this study we developed a 14-year land cover time series for the state of Mato Grosso, Brazil. MODIS NDVI and a spatiotemporally extensive ground reference dataset were utilized to develop a random forest classification model that was used to map the entire state for the 2001–2014 crop years. Annual INPE PRODES forest and Canasat sugarcane coverages were processed and incorporated into the maps, along with static urban and water information.

We found a more abrupt reduction of the deforestation rate in MT immediately following implementation of SoyM than has been reported in previous studies, consistent with claims that SoyM has played a role in reducing pressure to deforest [23,24,38]. In MT we observed a 5.7-fold decrease in annual deforestation rate post-SoyM (CY2007-2014) compared to pre-SoyM (CY2001-2006). We also observed a marked decline in forest-to-soy conversion rate post-SoyM compared to pre-SoyM, another expected outcome of SoyM. Looking at 1 to 5-year lags following deforestation, deforested areas ended up as soy at a rate 2.4 times greater pre-SoyM than post-SoyM. These findings suggest that the policy is helping eliminate the incentive to eventually use newly deforested lands for soybean production. However, the situation remains complex; in the property-level analysis of [24], the authors show that hundreds of “soy properties” in MT experienced post-SoyM deforestation in violation of Brazil’s Federal Forest Code, though they remained in compliance with SoyM because they did not plant soy in the deforested areas.

By analyzing total production increases attributable to vertical and horizontal intensification, we can account for the fact that mechanized agriculture may expand its *area* and *yields* both on recently deforested land and on other non-forest areas, especially existing cropland [39]. Focusing on soy, we found a pronounced increase in vertical cropping intensification over the study period. While our maps indicate that total soybean plantings have increased by a factor of 2.4 between CY2001 and CY2014, the total number of commercial crops harvested from soybean fields has increased by a factor of 3.5 due to increased use of double cropping. In terms of total number of commercial crops per soy pixel, we find this number has increased from 1.1 in CY2001 (indicating single crop soybeans to be the largely dominant management practice) to 1.6 in CY2014 (indicating that the majority of soybean crops are now being followed by a second commercial crop in the same growing season).

We found that soy producers in MT are intensifying production on existing soy fields (vertical) at 2.8 times the relative rate that they are expanding soy production to areas deforested before SoyM (horizontal). Understanding how this rate varies across space, with municipalities, watersheds, vegetation zones, etc. as the units of analysis, allows for exploring both the human and environmental dynamics that bring about intensification. A number of location-specific factors will also warrant consideration, such as production potential (agricultural aptitude) and market access, in addition to local and regional yield trends (e.g. MT soy, corn, and cotton yields from IBGE have increased by 0.3% yr^-1^, 4.5% yr^-1^, and 0.4% yr^-1^, respectively, where these values are the 14-year linear trend slopes divided by the 14-year average yields).

Our land cover map data are provided with this article (S3 Dataset). This is the first time such a detailed and extensive land cover dataset is available to the public covering this region of the world that has attracted so much attention concerning both the development of agriculture there and the policies designed to save remaining tropical forests. Policy researchers, land change scientists, non-governmental and governmental agencies, and the public can now take advantage of this reliable land cover dataset for various applications without having to produce the data themselves.

## Supporting information

S1 Fig Annual deforestation in the Amazon biome portion of Mato Grosso. Series from three independent studies are shown, all derived from PRODES data. Results from this study are most consistent with a large, direct SoyM effect on Amazon deforestation decline.
(TIF)

S2 Fig PRODES anomaly cleanup. Examples of PRODES forest ‘NoData’ cleanup are shown in the upper panels, whereas examples of PRODES ‘FORerr’ (bogus FOR pixels) cleanup are shown in the lower panels. Example locations are shown in the Mato Grosso map in the center, along with the Landsat tile overlap area and reduced area that was inspected during ‘FORerr’ cleanup.
(TIF)

S1 Supporting Information Primary supporting information file. All supporting information textual components referenced in the manuscript are provided in this document.
(PDF)

S1 Dataset Ground reference data. Crop year, land cover class, data source, and MODIS NDVI profiles are provided in an Excel spreadsheet for all 2681 samples comprising the ground reference dataset.
(XLSX)

S2 Dataset Roadside data. Crop year and land cover class are provided in a point shapefile for all 3185 samples comprising the roadside dataset.
(ZIP)

S3 Dataset Land cover map data. Final land cover maps for CY2001-2014 are provided for the state of Mato Grosso, Brazil.
(ZIP)
