# Supplementary figures and images for: Soy moratorium impacts on soybean and deforestation dynamics in Mato Grosso, Brazil
Source: PLoS One. 2017 Apr 28;12(4):e0176168. doi: 10.1371/journal.pone.0176168 (PMC5408992; doi:10.1371/journal.pone.0176168)

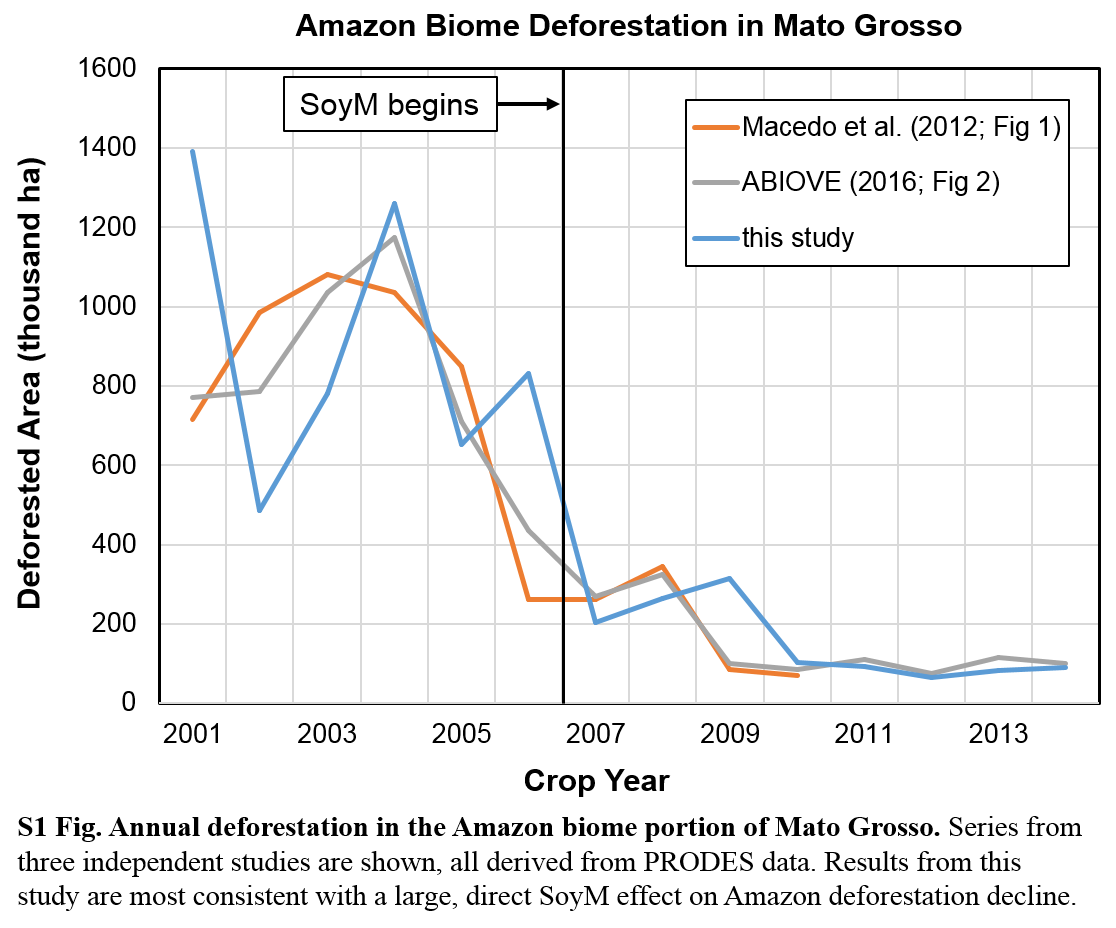

Supplement: S1 Fig — Series from three independent studies are shown, all derived from PRODES data. Results from this study are most consistent with a large, direct SoyM effect on Amazon deforestation decline. (TIF) [file pone.0176168.s001.tif]

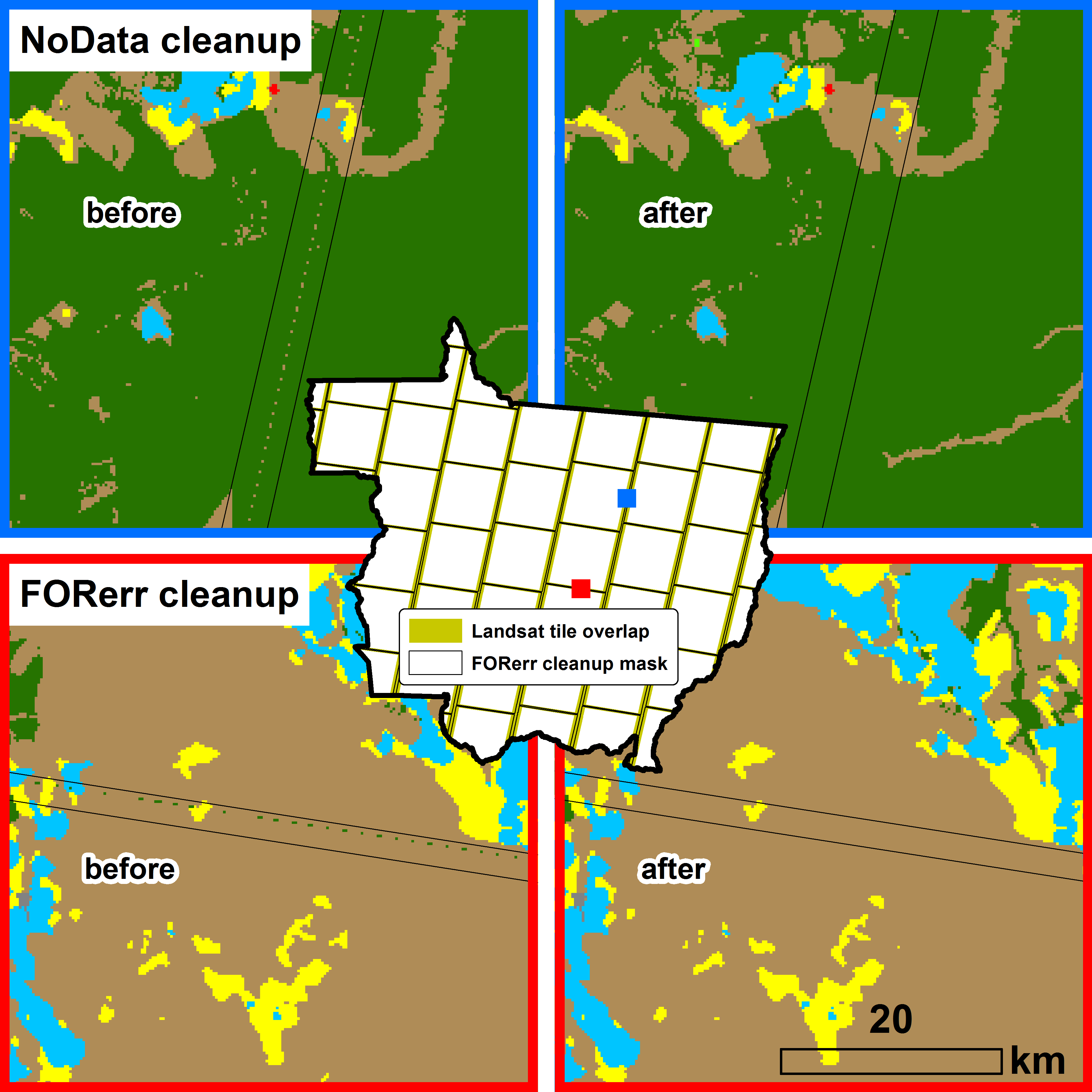

Supplement: S2 Fig — Examples of PRODES forest ‘NoData’ cleanup are shown in the upper panels, whereas examples of PRODES ‘FORerr’ (bogus FOR pixels) cleanup are shown in the lower panels. Example locations are shown in the Mato Grosso map in the center, along with the Landsat tile overlap area and reduced area that was inspected during ‘FORerr’ cleanup. (TIF) [file pone.0176168.s002.tif]
