# Supplementary material for: Soy moratorium impacts on soybean and deforestation dynamics in Mato Grosso, Brazil
Source: PLoS One. 2017 Apr 28;12(4):e0176168. doi: 10.1371/journal.pone.0176168 (PMC5408992; doi:10.1371/journal.pone.0176168)
Supplement: S1 Supporting Information — All supporting information textual components referenced in the manuscript are provided in this document. (PDF) [file pone.0176168.s003.pdf]

Supporting Information

A. Statement on the use the Random Forest (RF) model

In [1] we evaluated a boosted decision tree (DT) model for classification of the CY2005-2009 portion of the ground reference data used in this study. One of the conclusions was to consider RF as an alternative to DT modeling. In the early course of this research, we developed both boosted DT and RF models to determine if one approach was superior for our mapping needs. While cross validation accuracies for the two models were found to be somewhat similar when evaluated using pure pixel (endmember) ground reference data, visual inspection of classified maps developed using the two models suggested clear superiority of the RF model. The most visually evident improvement was observed along field boundaries, which consist largely of mixed pixels that frequently prove troublesome for models developed using endmember datasets. Draping the maps over high resolution imagery, the RF model appeared to appropriately classify field boundary pixels with much greater frequency than the boosted DT model. This observation was corroborated by a substantially improved correspondence with statewide annual IBGE soybean area estimates for the RF model output (described in the main text) compared to the boosted DT output, which tended to severely underestimate MT soy area. As a consequence of these observations, we decided to employ RF modeling.

B. Ground reference data preparation

Original land cover designations were recoded to one of five possible classes [1]: (1) pasture/cerrado; (2) soy-single (single crop soybeans, possibly followed by a cover crop); (3) soy-double (double crop soybeans, or soybeans followed by a commercial crop, excluding

cotton); (4) cotton; and (5) soy-cotton. According to IBGE statistics, crop classes (2)-(5) accounted for more than 95% of MT agricultural land area in CY2014.

The relatively few samples not fitting into one of these bins were discarded, such as rice and single crop corn. According to IBGE statistics, the discarded data were from areally minor cover types that did not fit into our class structure. An 85-15 filter [1] was used to purify the ground reference crop data, whereby all samples with at least 12 of 23 annual time series NDVI values falling outside the 15-85% class-specific and period-specific NDVI value percentile interval (i.e. the 70% data band) were excluded from the ground reference dataset. The excluded data represented severe statistical outliers presumed to be unreliable entries in our endmember ground reference dataset that was based on farmer records and recollections [1,2]. This statistical filter was not applied to pasture/cerrado samples from the two field campaigns, which were so small in number that a supplemental pasture/cerrado ground reference dataset was needed to increase sample size and to round out statewide representability of the pasture/cerrado data in support of the RF modeling effort.

Ideally our ground reference data, which we use for model development and accuracy evaluation, would include samples from all four of MT's main growing regions as identified in [3]. This would be more consistent with the principals of probability sampling with respect to spatial and areal representativeness of our mapped classes, which is an important consideration when evaluating map accuracy [4]. In reality, however, time and resource availability constrained our opportunistic, farmer interview-based ground data collection efforts, especially considering the immense size of our study area. With our available resources, it simply was not possible to reach all the growing regions during the two field campaigns. Though the state is

developing quickly, there are still only a relatively small number of reliable and easily trafficable roads.

Nevertheless, we know from interviews conducted in 2013 with representatives of major growers associations (APROSOJA, MT's Soy and Corn Growers Association; and FAMATO, MT's Agriculture and Ranching Federation) that our agricultural ground reference data are representative of the major classes and class proportions found throughout all of MT's main growing regions, with respect to growing season timing as well as the types of cropland management practices evident across the landscape. This assessment is corroborated by the favorable results obtained from the roadside data evaluation in the main text, which uses a substantially more spatially extensive set of reference points than the farmer interview-based ground reference data that were used for classification model construction.

C. Supplemental pasture/cerrado data acquisition

High resolution imagery was used to facilitate identification of additional pasture/cerrado ground reference samples from 82 locations around MT for CY2005-2009. This occurred in two separate efforts. The purpose of the first pasture/cerrado data supplementation was to increase sample size and mitigate underrepresentation of the pasture/cerrado data in the ground reference dataset from the field campaigns. The purpose of the second pasture/cerrado data supplementation was to improve the statewide representativeness of the NDVI profile distribution for this diverse, widespread and prominent cover type.

Locations for supplemental pasture/cerrado data samples were identified using the ESRI World Imagery layer (which at the time featured imagery from 2005) and Google Earth (2002-2011). This dataset provided needed areal and distributional enrichment of the pasture/cerrado ground reference data used for RF model development. Similar to [3], we employed

interpretation of aerial imagery to determine locations for supplemental pasture/cerrado ground reference data. When using visual inspection to obtain ground reference data, it is important to use imagery with higher spatial resolution than that of the finished classified map product [4]. To this we would add that using visual inspection to obtain ground reference data should only be done when it can be performed with high confidence, which requires that the cover type in question be visually (spectrally) distinct in the higher resolution imagery.

Areas covered by PRODES forest or IBGE water and urban layers were avoided. A visual assessment was conducted to identify areas where no particular managed land use (such as cultivated agriculture) was evident. Large areas that were homogeneous in appearance and visually representative of pasture/cerrado land cover were identified around the state. From these areas, maximally interior points were placed and NDVI profiles extracted [5]. For larger tracts, multiple interior points were placed using approximately uniform spacing. NDVI profiles from CY2005-2009 for each point were examined for expected pasture/cerrado spectral behavior (phenology), and points exhibiting obviously inconsistent behavior (such as having the appearance of a crop profile) were eliminated from consideration. Following this spectral screening, a total of 276 points remained. To maintain appropriate class proportionality for modeling (which requires consideration of class separability and emphasis for model development), it was determined that 45 points would be randomly selected from this set and appended to the ground reference dataset. With five samples taken from each point (one for each crop year 2005-2009), this resulted in the addition of 225 pasture/cerrado samples.

Identification and remediation of visually evident high misclassification areas is a recommended practice for quality map construction; obvious errors in preliminary outputs should be identified and rectified if possible [4]. After we inspected a preliminary map series

developed using the ground reference data and the supplemental pasture/cerrado samples just described, we determined that managed pasture locations were frequently misclassified as soy-single. Managed pastures have a distinct appearance in high resolution imagery, and they should fall in the pasture/cerrado class. To mitigate this problem, 22 managed pasture points, along with 15 additional cerrado points from other visually evident high misclassification areas around the state, were added to improve statewide pasture/cerrado representation in the ground reference dataset. This process was completed with the same protocol used for the first supplemental pasture/cerrado extraction. With data extracted from CY2005-2009 for these new sites, this resulted in the addition of 110 managed pasture and 75 cerrado samples. Thus, in total across both supplemental pasture/cerrado data selection efforts, 410 pasture/cerrado samples were added to the ground reference dataset.

Pasture/cerrado sample count values shown in Table 1 reflect the full pasture/cerrado ground reference dataset, which includes the samples from both supplementation efforts in addition to the samples obtained during the two field campaigns. Fig 1 shows the point locations for the supplemental pasture/cerrado sites, and the top-right panel in Fig 2 shows spectral profile statistics for all the ground reference samples in the pasture/cerrado class. As evident from the pairwise Jeffries-Matusita (JM) distance statistics shown in the top-left panel in Fig 2, the pasture/cerrado class generally exhibited a high degree of separability from the other mapped classes, which supports its smaller proportion of our total ground reference dataset compared to its proportion of total mapped class area in MT.

D. PRODES forest/deforestation data preparation and processing

The PRODES data consist of dated (year and day-of-year, or DOY) and classified polygons, of which four classes were used in this study: (1) DESFLORESTAMENTO, or

deforested, hereafter DEF; (2) RESIDUO, or residual deforested, hereafter RES; (3) FLORESTA, or forest, hereafter FOR; and (4) NUVEM, or clouds, hereafter NUV. PRODES files covering the MT study area were downloaded, merged, and clipped.

The FOR class (94,267 polygons totaling 31.1 Mha) denotes areas that remained as forest in some final inspected Landsat scene, all of which were dated from calendar year 2014. The NUV class (762 polygons totaling 88.5 thousand hectares, or Kha) indicates areas last known to be forest that were obstructed by clouds in the most recent annual PRODES classification, and which were thus unable to be examined for deforestation. Consequently, we reclassified NUV as FOR, which increased FOR area by 0.3%. The DEF class represents deforested area, with the provided date (year + DOY) representing the acquisition date for the first Landsat scene where deforestation was detected. The RES class also represents deforested area, but with greater uncertainty regarding when deforestation occurred. So, while we can assume that the Landsat scene acquisition date for DEF records is reasonably representative of the date of deforestation, for RES records we only know that this area was deforested at some previous time that may or may not be close to the associated Landsat scene acquisition date. Despite this uncertainty, we treated RES records identically to DEF records, reasoning that because RES polygons were known to be forest at some previous time during the PRODES period of record, the best guess for the deforestation date is the provided date. Later we discuss the potential impact of this assumption on the analysis.

To construct the annual (crop year) forest extent maps, we first developed the map for CY2014 and then worked backward in time by adding forest area to the previously processed year's forest extent. To obtain the CY2014 forest coverage, DEF/RES polygons dated 2013(day 209) or later were merged with the FOR polygons. To obtain forest cover for CY2013, DEF/RES

polygons dated between 2012(day 209) and 2013(day 208) were added to the CY2014 forest set, and so on working back to CY2001.

In the study area, the three targeted classes (DEF, RES, FOR) comprised 329,469 PRODES polygons totaling 39.7 Mha. The following four complications regarding the PRODES data required redress before processing:

1) How to handle DEF/RES records that lacked a DOY value ('0-DOY' records)
2) How to handle records from the RES class
3) How to handle map pixels lacking PRODES coverage ('NoData' pixels)
4) How to handle bogus FOR ('FORerr') map pixels occurring in Landsat tile overlap regions

Details and method for handling:

1) 9909 DEF/RES polygons totaling 361.6 Kha had a 0 value for DOY. All such 0-DOY records occurred in calendar years 2005, 2013, and 2014, with the vast majority occurring in 2005. Supplemental Landsat acquisition date information provided to the authors by INPE in a personal communication (13-Jan-15) resolved all of the 2005 problems. 214 polygons (2630 ha) with 0-DOY remained from calendar year 2013, which were all classified as RES. Considering that 74% of the 224,531 unaffected DEF/RES polygons (and 75% of the unaffected DEF/RES total area) had DOY > 208, we decided to assign all of the 0-DOY polygons from 2013 to CY2014 (meaning they would remain as FOR in the final map for CY2014). Regardless of the validity of this handling, the small affected area ensures a marginal impact on the analyses.

2) RES records consisted of 29,325 polygons totaling 334.5 Kha. Recall that these polygons were identified as being deforested at some previous, unspecified point in time during the PRODES period of record, but not necessarily near the associated Landsat scene acquisition date. We decided to treat these polygons identically to the DEF polygons and associated them with their Landsat scene date, with this treatment expected to be more reasonable than leaving them as either forest or non-forest throughout the study period. 70% (233.7 Kha) of the RES records occur pre-SoyM (CY2001-2006) and 30% (100.8 Kha) occur post-SoyM (CY2007-2014). The pre-SoyM RES total accounts for 3.7% of the total pre-SoyM deforested area (= DEF+RES = 6.3 Mha deforested during CY2001-2006). The post-SoyM RES total accounts for 7.3% of the total post-SoyM deforested area (= DEF+RES = 1.4 Mha deforested during CY2007-2014). With these relatively small proportions of the overall deforested area pre- and post-SoyM, the effect of this approach to RES record handling on our deforestation analyses examining differences between pre-SoyM and post-SoyM time periods will be minor.

3) The PRODES dataset harbors a number of ‘stich line’ data gaps predominantly occurring in Landsat tile overlap zones (the PRODES coverage was developed primarily using Landsat data). In total, upon conversion to the 240-m MODIS grid, there were 5418 ‘NoData’ MODIS pixels. For each ‘NoData’ pixel and each year, if the majority (>50%) of its (typically) eight-pixel set of immediately adjacent or diagonal neighbors were FOR, then the missing pixel was classified as FOR. For complete application of this strategy, the process was repeated until no more pixels were changed, which required four iterations. With the RF classification providing the default assignment for the ‘NoData’ pixels, a total of 1085 unique pixels were affected by the neighborhood analysis, resulting

in 13,662 FOR assignments during CY2001-2014, for an average of 12.6 recoded years per pixel out of a possible 14. This high per-pixel rate of recode suggests that most of the adjusted pixels were interior to stable forest regions. An example location before and after treatment is shown in the upper panels of S2 Fig.

4) Visual inspection of preliminary MT land cover maps revealed a number of 'stitch line' forest artifacts in non-forest areas. These bogus FOR ('FORerr') pixels were found to occur exclusively within central portions of Landsat tile overlap zones. Though strips of offending PRODES polygons generally were apparent to the eye, their distribution was not sufficiently unique in size, shape, or attribution to allow for clean isolation from the source data. Instead, a spatial filtering approach was developed and applied to each year of the annual LULC map set to mitigate the problem. First, visual inspection was used to reduce the Landsat overlap region (described in S6) to minimize the search area while still including all visually apparent FORerr pixels. All FOR pixels interior to the mask area were examined, and those with at least seven non-FOR neighbors (from a possible eight) were reassigned to their RF class. With FOR a declining coverage over time, the number of adjustments generally increased over time, with 1193 FOR pixels overwritten in CY2001 and 1963 FOR pixels overwritten in CY2014. A second iteration was applied whereby reassignment required all eight neighboring pixels to be non-FOR, which resulted in a small number (ranging from 57 to 80) of additional FOR pixels being overwritten each year during CY2001-2014. In total, annual recoded area ranged from 7,229 ha in CY2001 to 11,866 ha in CY2014. An example location before and after treatment is shown in the lower panels of S2 Fig.

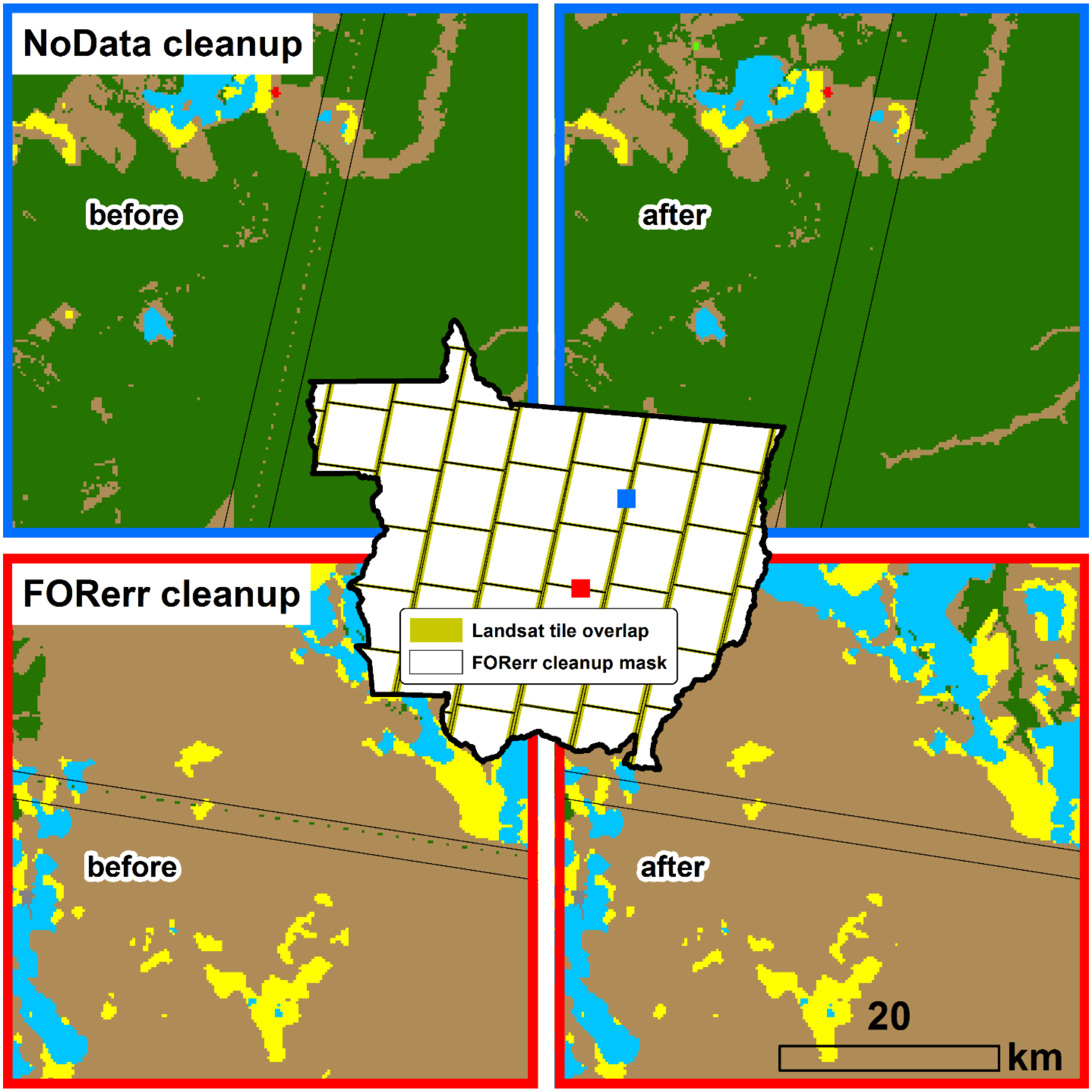

S2 Fig. PRODES anomaly cleanup. Examples of PRODES forest 'NoData' cleanup are shown in the upper panels, whereas examples of PRODES 'FORerr' (bogus FOR pixels) cleanup are shown in the lower panels. Example locations are shown in the Mato Grosso map in the center, along with the Landsat tile overlap area and reduced area that was inspected during 'FORerr' cleanup.

E. Backfilling missing years of Canasat sugarcane data

INPE Canasat data, which were kindly provided by the dataset stewards for the development of this work, were used to define annual sugarcane coverages for MT for CY2003 and CY2005-2014. The INPE MODIS visualization tool [6] and professional judgment were used to characterize CY2003 and CY2005 Canasat sugarcane fields as either 'sugarcane' or 'not sugarcane' in the missing Canasat crop years of 2001, 2002, and 2004, with fields of the latter class opened to classification using the RF model.

Unlike the crop types used in the RF model, due to sugarcane's unique management and frequently irregular phenology (e.g. 12 and 18-month crop cycles are common among sugarcane management practices in MT), NDVI profiles from sugarcane fields often are highly variable and thus exceptionally difficult to consistently and correctly classify in a standard, single-year fashion. Consequently, we elected to apply a subjective visual inspection approach (whereby multiple consecutive years of NDVI profiles can be examined at once) to classify the missing years of sugarcane information rather than attempting to develop a deterministic model that assumes somewhat consistent annual phenology.

Considering the relatively small (4% of the total cropland area) and spatially invariant (Fig 3) areas that were affected, this manual approach provided our best option with respect to reasonability (once established, sugarcane tends to be continuously cropped rather than rotated to other, non-sugarcane lands) and practicality (sugarcane often has an irregular, multi-year phenology pattern due to its management, which greatly complicates its mapping in the traditional annual profile endmember framework). This sugarcane backfill effort impacted just 3 of the first 4 study years, when sugarcane area in MT was the lowest during the study period (Table 3).

The CY2004 sugarcane coverage was initialized by including all areas that appeared in both CY2003 and CY2005 Canasat datasets. Next, the remaining sugarcane areas appearing in either CY2003 or CY2005 were examined using the Temporal Vegetation Analysis System MODIS visualization tool (SATVeg; www.satveg.cnptia.embrapa.br). Professional judgment was used to characterize some of these areas as sugarcane in CY2004, with areas not identified as sugarcane assigned to their RF class. NDVI profiles from CY2002 were examined for the CY2003 Canasat areas and handled in the same manner, with areas identified as sugarcane in CY2002 further examined to define the CY2001 sugarcane coverage. With this approach to filling in missing Canasat years, we could ensure both spatial consistency with the Canasat dataset while also exhibiting the increase in sugarcane area known to exist in MT during the study period.

F. Processing details for step #3 of map development

The following processing steps were applied to "Identify and repair PRODES data commission and omission anomalies" (step 3a in the text):

1) Fill in missing FOR pixels:
    a. Identify instances of non-FOR before FOR, anomalous behavior attributable to rasterization of overlapping polygons (topology errors) in PRODES
    RESULT: 1633 instances were identified from 324 unique pixels, all of which were reclassified as FOR.
    b. Identify and repair PRODES 'NoData' pixels (results described in S4)
        i. Year-by-year, recode to FOR if majority of immediate neighbors are FOR
        ii. Repeat (i) until convergence (four iterations were required)

2) Remove FORerr pixels (eliminate all FOR islands of 1, 2, or 3 pixels in a horizontal or vertical line, within a narrowly focused portion of the Landsat tile overlap zone):
   a. Create Landsat tile overlap coverage, and through trial and error, systematically reduce its size as much as practical to keep all visually obvious FORerr pixels enveloped RESULT: An 8 km inward buffer was used for the vertical overlap regions, and a 3 km inward buffer was used for the horizontal overlap regions (see MT map in S2 Fig). The final mask contained 4.5% of the total MT area, including 3.7% of the total MT forest area for each year.
   b. [filter pass one] Year-by-year, for each FOR pixel in the mask, if at least 7 of its 8 neighbors were non-FOR, then recode to Step 1 RF class (results described in S4)
   c. [filter pass two] Year-by-year, for each FOR pixel in the mask, if all 8 neighbors were non-FOR, then recode to Step 1 RF class (results described in S4)

The following processing steps were applied to "Identify and repair illogical pixel LC trajectories" (step 3b in the text):

1) Create final dataset by applying the following pixel-level adjustments to unlikely temporal trajectories (* = crop class; P = pasture/cerrado; F = forest; trajectories include either 3 or 4 consecutive years of LULC information, with the '*' appearing in the year of adjustment):
   a. CY2001: Recode instances of *PP to PPP
   b. CY2002: Identify instances of P*P and F*P and replace * with P
   c. CY2003-2014 (see NOTE below for handling of CY2014): Identify instances of PP*P, FP*P, and FF*P and replace * with P

d. Repeat (a)-(c) until convergence (six iterations required)

   RESULT: In total across all 14 years, 830,882 recodes were applied to 618,471 pixels (3.9% of the study area). On average, 0.4% of the study area (5.5% of the cropland area) was adjusted each year, with the fewest changes occurring in CY2004 (22,661 pixels, or 2.9% of the cropland area) and the most occurring in CY2011 (136,364 pixels, or 10.3% of the cropland area).

NOTE: Though we classified MODIS through CY2015 to use for CY2014 trajectory adjustments, we did not have complete ancillary data for CY2015, nor were we able to properly inspect classification results from CY2015 for land cover trajectory anomalies as described above. Consequently, mapping results from that year are not included in the study.

References

1. Brown JC, Kastens JH, Coutinho AC, Victoria DC, Bishop CR. Classifying multiyear agricultural land use data from Mato Grosso using time-series MODIS vegetation index data. Remote Sensing of Environment. 2013; 130:39-50. doi: 10.1016/j.rse.2012.11.009

2. Brown J, Jepson W, Kastens J, Wardlow B, Lomas J, Price K. Multitemporal, Moderate-Spatial-Resolution Remote Sensing of Modern Agricultural Production and Land Modification in the Brazilian Amazon. GIScience & Remote Sensing. 2007; 44(2):117-148. doi: 10.2747/1548-1603.44.2.117

3. Arvor D, Meirelles M, Dubreuil V, Bégué A, Shimabukuro YE. Analyzing the agricultural transition in Mato Grosso, Brazil, using satellite-derived indices. Applied Geography. 2012; 32(2):702-713. doi: 10.1016/j.apgeog.2011.08.007

4. Olofsson P, Foody GM, Herold M, Stehman SV, Woodcock CE, Wulder MA. Good practices for estimating area and assessing accuracy of land change. Remote Sensing of Environment. 2014; 148:42-57. doi: 10.1016/j.rse.2014.02.015

5. Wardlow BD, Egbert SL, Kastens JH. Analysis of time-series MODIS 250 m vegetation index data for crop classification in the U.S. Central Great Plains. Remote Sensing of Environment. 2007; 108:290-310. doi: 10.1016/j.rse.2006.11.021

6. Freitas RM, Arai E, Adami M, Souza AF, Sato FY, Shimabukuro YE, et al. Virtual laboratory of remote sensing time series: visualization of MODIS EVI2 data set over South America. Journal of Computational Interdisciplinary Sciences. 2011; 2(1):57-68. doi: 10.6062/jcis.2011.02.01.0032
